# Carbolic Acid

**Published:** 1883-01

**Authors:** 


Carbolic Acid.—There is no therapeutical agent so extensively used among dentists as carbolic acid, and in by far the greater proportion of cases it is undoubtedly misapplied. It makes up the entire pharmacopœia of many practitioners. It is applied in septic and aseptic conditions, to hyperæmic and anæmic tissue. It is thrust into the nerve canals of offensive, devitalized teeth, and it is freely applied to exposed and living pulps. It is pumped into alveolar abscesses, and it is daubed upon hypertrophies. Hard tissues and soft tissues, tooth, bone, cartilage, muscle and mucous-membrane, all receive the same impartial treatment. In fact many supposed intelligent dentists make of it a universal specific, and trust to it to cure every oral disease that they meet with in practice.

Dentists, no more than any other class of practitioners, have the right to prescribe blindly. Unless they know the nature and office of a drug, it is trifling with life and health for any one to use it, and the application of irritants to inflamed and angry tissue indicates recklessness of results as well as ignorance of methods. Carbolic acid is a local irritant, and when applied to soft tissues forms a white eschar, which is only removed by the usual process of sloughing. When, therefore, this delicate tooth-pulp in cases of simple exposure is seared with it, and a cap of any material is placed over it, there is in contact with the living tissue a scab of disintegrated material which the tooth has no means of eliminating. The very process of thus cauterizing a part of the tooth-pulp irritates the remainder and induces an angry condition, which the judicious operator would avoid.

The chief virtue in carbolic acid is its destructive influence upon the lower forms of vegetable and animal life. Through this property it arrests fermentation and acts as a powerful antiseptic, and this is its principal recommendation for dentists. If forced into a septic nerve-canal, it renders it aseptic; but, at the same time, if it reach, either by penetration or absorption, to the tissues beyond the apical foramen, great pain and inevitable inflammation are the consequence. There are other agents which bring about the desired results without these evil complications. We have frequently heard dentists complain that upon opening the pulp-chamber of a dead tooth which was slightly offensive but had hitherto been painless, after a bit of cotton lint dipped in carbolic acid or creosote had been forced into it, the most intense pericementitis has ensued, with pain so severe that the tooth was, perhaps, sacrificed for the comfort of the patient. What, then, was asked, was the cause of all this trouble? The answer must be plain to any reflecting practitioner. The remedy itself produced it. Why was an agent so drastic and irritating in its nature placed in contact with tissues already urged almost to the point of an outbreak? It was a misapplication of remedies.

Again, it is frequently pumped into a blind abscess, and the only means of exit for the sanious products is tightly plugged up with a pledget of cotton. What wonder that the effete matter (the albuminous portions of which have united with the agent, and being thus dammed up in a disturbed territory) should cause an increase of pain? It is but another instance of the misapplication of a remedy.

If applied to hyperæmic tissues, the inflammation is augmented. If to anæmic tissues, they are destroyed. Yet both are too often freely dressed with it. Are we not right when we affirm that as a therapeutic agent, as it is commonly used, a far greater amount of injury than benefit is the result? In view of all these facts, carbolic acid and creosote have for some time been regarded by us with a constantly growing disfavor, until at the present time their employment in our operating room has become very rare indeed. Creosote was some years since banished entirely from the medicine case, and carbolic acid is used almost solely for its local anæsthetic properties. When it becomes necessary to excise soft hypertrophies, a free use of this remedy upon the surface will nearly obtund feeling to a considerable depth. It is also sometimes useful in destroying or diminishing the pain arising from the cutting of sensitive dentine.

As an antiseptic, a solution of salicylic acid is the more powerful, while it is destitute of those offensive qualities which make carbolic acid so objectionable. For cauterizing, a solution of nitrate of silver is much preferable. But the preparation which has largely superseded carbolic acid in our practice is oil of eucalyptus, or eucalyptol. Lacking the irritating qualities of the former, it is quite as effective in treating septic nerve canals. A tooth containing a dead pulp may be freely dressed with it without fear of bringing on an active stage of pericementitis. Indeed, it has been found useful in those painful conditions, and has brought about a resolution when other agents failed.

In cases of irritable, inflamed and congested gums, when carbolic acid has not infrequently been improperly used, a far better remedy is chloride of zinc, injected beneath the turgid tissue and into the pockets formed at the sides of the teeth. A few applications of this will relieve the congested condition, and make practicable the removal of deeply-seated deposits, which are often the source of the trouble.

